# Possible involvement of l-arginine-nitric oxide pathway in the antidepressant activity of Auraptene in mice

**DOI:** 10.1186/s12993-022-00189-1

**Published:** 2022-02-14

**Authors:** Hossein Amini-Khoei, Shakiba Nasiri Boroujeni, Forough Maghsoudi, Mohammad Rahimi-Madiseh, Elham Bijad, Mohammadtaghi Moradi, Zahra Lorigooini

**Affiliations:** Medical plants Research Center, Basic Health Sciences Institute, Shahrekord University of Medical Sciences, Shahrekord, Iran

**Keywords:** Depression, Nitric oxide, Auraptene, Coumarin derivative

## Abstract

**Background:**

Depression is one of the most common mental illnesses worldwide. Nitric oxide (NO) is involved in the pathophysiology of depression. Auraptene (a coumarin derivative) has been shown to possess pharmacological effects on neurological diseases.

**Purpose:**

The present study aimed to investigate the possible role of the NO pathway in Auraptene antidepressant effects in male mice.

**Methods:**

Behavioral tests were used to assess depression-like behaviors. The mice received Auraptene at 10, 30, and 100 mg/kg, the combination of the sub-effective (ineffective) dose of Auraptene (10 mg/kg) and L-NAME, and the combination of the effective dose of Auraptene (30 mg/kg) and L-arginine. Finally, OFT, TST, FST, brain, serum MDA level, antioxidant capacity, hippocampus, and serum NO level were measured.

**Results:**

The data analysis showed that Auraptene (30 mg/kg) improved depression-like behaviors. Auraptene (30 mg/kg) also significantly reduced serum NO levels (P < 0.05) and significantly increased serum MDA (10 mg/kg, P < 0.05). Auraptene at 30 mg/kg also increased serum antioxidant capacity (P < 0.01). Co-administration of L-NAME and the sub-effective dose of Auraptene enhanced the effects of Auraptene. However, co-administration of the effective dose of Auraptene and L-arginine reduced the impacts of Auraptene.

**Conclusions:**

The results showed that Auraptene causes antidepressant effects in a dose-dependent manner and acts as a prooxidant at 100 mg/kg, and exacerbates oxidative stress. The antidepressant effects of this active molecule are exerted by reducing the NO level in the hippocampus and serum, increasing the antioxidant capacity, and reducing the MDA level in the serum.

## Introduction

Depression is one of the most common psychiatric diseases with a prevalence of 15-25% and can cause a significant decline in patient performance in all occupational areas and social and family relationships [1]. Decreased function of neurotransmitters such as serotonin (5-HT), norepinephrine or noradrenaline (NA), and dopamine (DA) are some of the causes of depression [2].

Oxidative stress is an influential factor for various central nervous system disorders and can accelerate the aging process and lead to behaviors related to depression and anxiety. During oxidative stress, the production of free radicals exceeds the capacity of the body’s antioxidant defense system, including enzymatic components (such as catalase) and non-enzymatic components (such as vitamin C and vitamin E) to neutralize them. Consequently, free radicals cause cell damage and death by attacking various components, including nucleic acids, proteins, enzymes, and cell membrane lipids [3]. Serum malondialdehyde (MDA) level is one of the markers of lipid peroxidation and the critical indicator to evaluate oxidative stress. It has been explained that the total antioxidant capacity of serum is significantly reduced in people with major depressive disorder [4].

Nitric oxide (NO) is a free gas and messenger molecule that regulates the nervous and immune systems. Some studies have shown that NO is involved in depression and stress [5]. There are three different genetic isoforms of nitric oxide synthase [6] for NO production, including neuronal nitric oxide synthase (nNOS), endothelial nitric oxide synthase (eNOS), and induced nitric oxide synthase (iNOS). Several studies have shown the role of nNOS in the pathophysiology of depression, and antidepressants have been shown to reduce NO levels in the patient’s serum [7–9].

Pharmacotherapy for depression can be associated with unwanted side effects such as anticholinergic and arrhythmic effects, which increasingly intensify the need to identify common drugs used to treat depression [10, 11]. Medicinal plants are a rich source of active secondary metabolites that widely draw researchers’ attention [12].

Auraptene (7-geranyloxycoumarin) is a coumarin derivative isolated from the skin of some citrus and is the most abundant form of natural geranyloxycoumarin. Auraptene is found in many plants of the citrus genus, such as grapefruit and oranges [13], and has valuable medicinal properties, including anti-cancer, antibacterial, antifungal, anti-inflammatory, and antioxidant. Auraptene is also known to protect the nervous system [14–17].

Some coumarin compounds isolated from plant species exert inhibitory effects on monoamine oxidase. Monoamine oxidase A (MAO-A) selectively targets the catalysts of the neurotransmitters of serotonin and norepinephrine and is a pharmacological target in seeking out beneficial agents for the treatment of depression [18]. The evidence shows that coumarin compounds inhibit TNF-α or PGE2 secretion by affecting NFkB nucleus transport and inhibiting the phosphorylation of P38, JNK1/2, PKC kinases in LPS-stimulated macrophages and mononuclear cell lines. Moreover, research has shown that all coumarin compounds affect NO production by reducing the expression and activity of the iNOS gene and its protein, indicating the anti-inflammatory activity of coumarin compounds, e.g., Auraptene [19]. Coumarin compounds have been shown to exhibit anti-cancer properties by downregulating the PI3K/Akt and MEK/ERK pathways and increasing P-gp expression [20].

Therefore, this study aimed to investigate the antidepressant effects of Auraptene in male mice concerning the role of the NO pathway.

## Materials and methods

### Study design

80 male NMRI mice were divided into eight groups (n = 10) as follows [21–23]:

1. The group received normal saline.
2. Experimental group received Auraptene at the dose of 10 mg/kg.
3. Experimental group received Auraptene at the dose of 30 mg/kg.
4. Experimental group received Auraptene at the dose of 100 mg/kg.
5. The group received nitric oxide synthetase [6] inhibitor (L-NAME) at 10 mg/kg.
6. The group received L-arginine (L-arg) at 100 mg/kg 45 min before the test.
7. The group received a sub-effective dose of Auraptene plus L-NAME.
8. The group received an effective dose of Auraptene plus L-arg.

Auraptene was injected acutely and simultaneously with NO mediators intraperitoneally [21–23]. After injections, behavioral tests were performed, and after that, the brain and blood samples of mice under deep anesthesia with chloroform to minimize stress were taken. Then malondialdehyde content, antioxidant capacity, and NO in the brain and serum of mice were measured.

### Behavioral tests

#### Open field test (OFT)

This test is used to evaluate the stress and emotional stability of rodents. The experimental groups were assessed on PND 45 using an open field apparatus, a box made of Plexiglas with dimensions of 60 × 50 × 40 cm.

The box floor area was divided into 16 equal squares, and the movements of each mouse were monitored for 5 min. This test is also used to assess anxiety based on vertical and horizontal directions and the amount of body scratching [24].

#### Tail suspension test (TST)

To do TST, each mouse was suspended from the edge of a rod (50 cm above a tabletop) by adhesive Scotch tape located about 1 cm from the tip of the tail. Tail ascending was banned by passing the mouse’s tail through a small plastic cylinder before the suspension. The period of immobility was recorded for 6 min. Animals were considered immobile when they hung down passively and remained utterly motionless [25].

#### Forced swimming test (FST)

This test has been approved to investigate depression in rodents. According to the theory of helplessness of Martin Seligman, if the animal is exposed to constant stress and has no way out of it, it gradually loses hope of escaping and becomes immobile. The glass container (25 × 12 × 15 cm) was filled with 25 °C water, and the animal was gently placed in the water from a height of 20 cm. The discontinuation of movements of the mouse was considered immobility. The total duration of the FST is 6 min, 2 min for the animal’s adaptation, and 4 min for measuring immobility. Increasing the immobility duration is considered depression, and its decrease is regarded as the effectiveness of antidepressant treatment [26].

### Biochemical tests

#### Measurement of the brain and plasma malondialdehyde levels

A working solution was used for measuring the amount of malondialdehyde (MDA). It contained 0.5 g of thiobarbituric acid, 80 ml acetic acid 20%, whose pH reached 5.3 by adding sodium hydroxide (NaOH) using a pH meter, and its final volume was increased to 100 ml by the addition of 20% acetic acid. In the next step, 100 µl of the sample, 100 µl of 8.1% SDS, and 2.5 ml of the working solution were mixed in a glass test tube and then the tubes in boiling water for one hour. The tubes were then cooled and centrifuged at 4000 rpm. Then the optical absorbance of the supernatant was recorded at 523 nm [27].

#### Determination of serum and brain total antioxidant capacity

The basis of the FRAP method is the ability of serum to reduce Fe+3 ferric ions to ferrous Fe+2 in the presence of an agent called TPTZ. In this method, the Fe+2 reaction with the TPTZ reagent creates a blue Fe+2-TPTZ complex with a maximum absorbance of 593 nm. The serum’s reducing power was measured by increasing the concentration of the above complex using spectrophotometry [28].

#### Determining the nitric oxide level of the hippocampus

To determine the NO level in the hippocampus, nitrite, as a stable product of NO, was measured in different groups of animals after administration of different doses of Auraptene in the presence and absence of selective NOS inhibitor based on the Griess test. The basis of this reaction is the formation of the diazotization dye of a sulfonamide with the help of nitrite in an acidic medium and then its conjugation with an aromatic amine N-naphtyle ethylenediamine1 (NEDD) [22, 29].

#### Data analysis

Statistical analysis was performed using Prism software, and the results were expressed as Mean ± SEM. Data were analyzed by one-way analysis of variance (ANOVA) and Tukey’s post hoc test. The results were presented as mean plus SEM. P < 0.05 was considered a significance level.

## Results

### The number of horizontal movements in the OFT

**Fig. 1:**
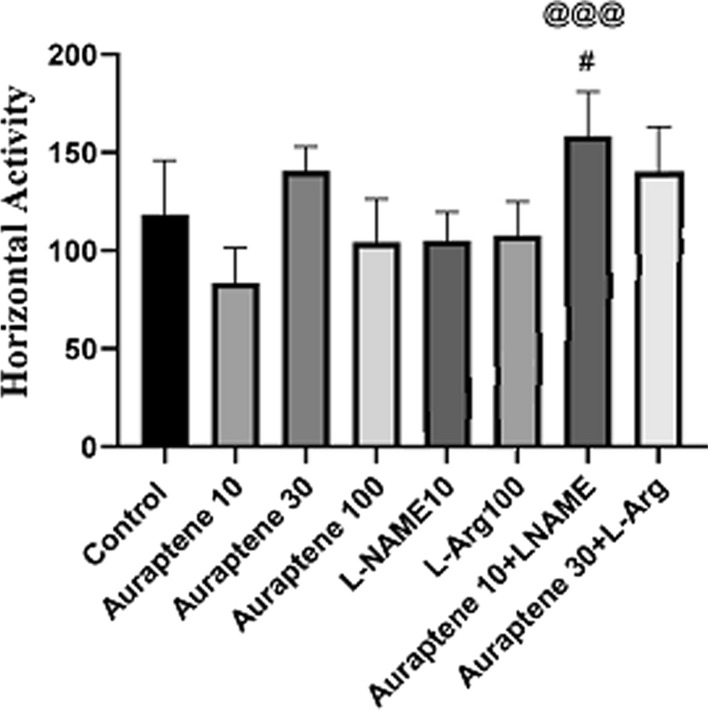
The amount of horizontal movements in OFT in the experimental groups. Data were reported as Mean ± SEM and analyzed using one-way ANOVA and Tukey post hoc test. ^#^ P < 0.05 in compared to the normal group. ^@@@^P < 0.001 in compared to the group that received Auraptene at a dose of 10 mg/kg

As illustrated in Fig. 1, co-administration of Auraptene (10 mg/kg) and L-NAME significantly increased the number of horizontal movements in the OFT compared to the normal saline group (P < 0.05), and the group received Auraptene at 10 mg/kg (P < 0.001). However, no significant difference was observed between the groups that received other doses of Auraptene in comparison with the normal group.

### The number of vertical movements in the OFT

**Fig. 2:**
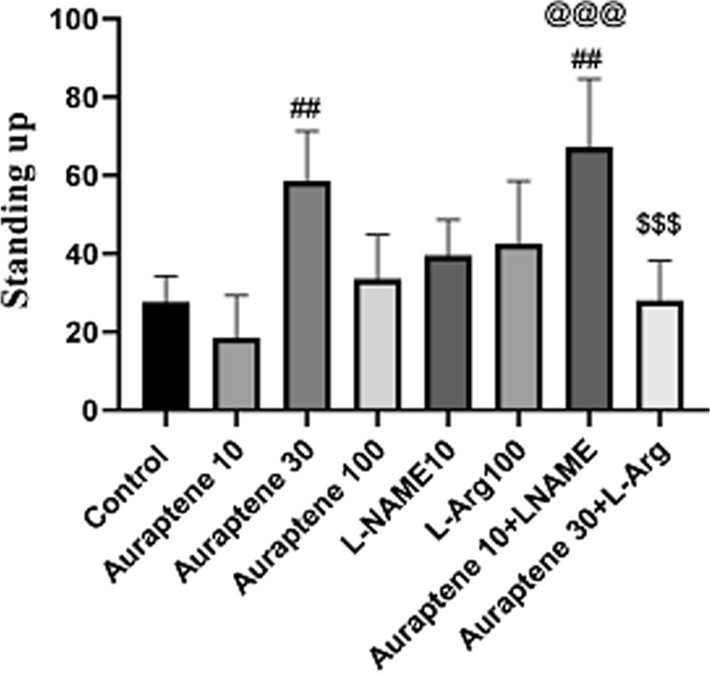
The amount of vertical movements in OFT in the experimental groups. Data were reported as Mean ± SEM and analyzed using one-way ANOVA and Tukey post hoc test. ^##^ P < 0.01 in compared to the normal group. ^@@@^ P < 0.001 in compared to the group that received Auraptene at a dose of 10 mg/kg. ^&&&^ P < 0.001 in compared to the group that received Auraptene at a 30 mg/kg dose

The one-way ANOVA and Tukey’s post hoc test (Fig. 2) showed that administration of Auraptene at 30 mg/kg significantly increased the number of vertical movements in the OFT compared to the normal saline group (P < 0.01). Moreover, co-administration of Auraptene (10 mg/kg) and L-NAME significantly increased this movement compared to the normal saline group (P < 0.01). Administration of 10 mg/kg Auraptene plus L-NAME significantly increased the number of vertical movements compared to the group received 10 mg/kg Auraptene alone (P < 0.001). Administration of Auraptene at a dose of 30 mg/kg plus L-Arg significantly reduced the number of vertical movements compared to the group that received 30 mg/kg Auraptene alone (P < 0.001). However, the administration of Auraptene at the doses of 10 and 100 mg/kg and L-NAME and L-Arg alone did not have any significant difference in comparison with the normal saline group.

### The amount of scratching in the OFT

**Fig. 3:**
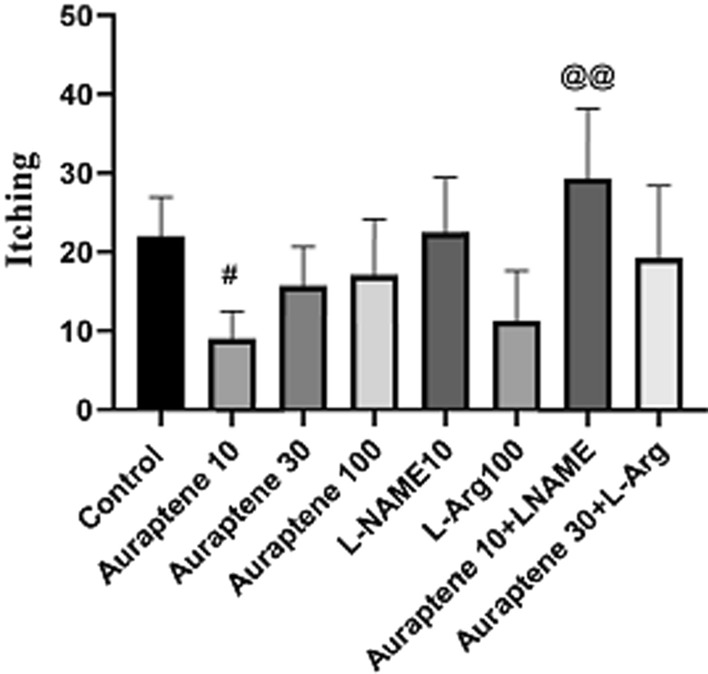
The scratching rate in OFT in the experimental groups. Data were reported as Mean ± SEM and analyzed using one-way ANOVA and Tukey post hoc test. ^#^ P < 0.05 in compared to the normal group. ^@@^P < 0.01 in compared to the group that received Auraptene at a dose of 10 mg/kg

The results of the amount of scratching in the OFT (Fig. 3) showed that the administration of Auraptene at doses of 30 and 100 mg/kg did not make any significant difference with the normal saline group. It should be noted that the administration of Auraptene at a dose of 10 mg/kg significantly reduced the amount of scratching in the OFT compared to the normal saline group (P < 0.05). Also, administration of Auraptene at a dose of 10 mg/kg plus L-NAME significantly increased the amount of this behavior is compared to the group that received 10 mg/kg Auraptene alone (P < 0.01).

### The duration of immobility in the TST

**Fig. 4:**
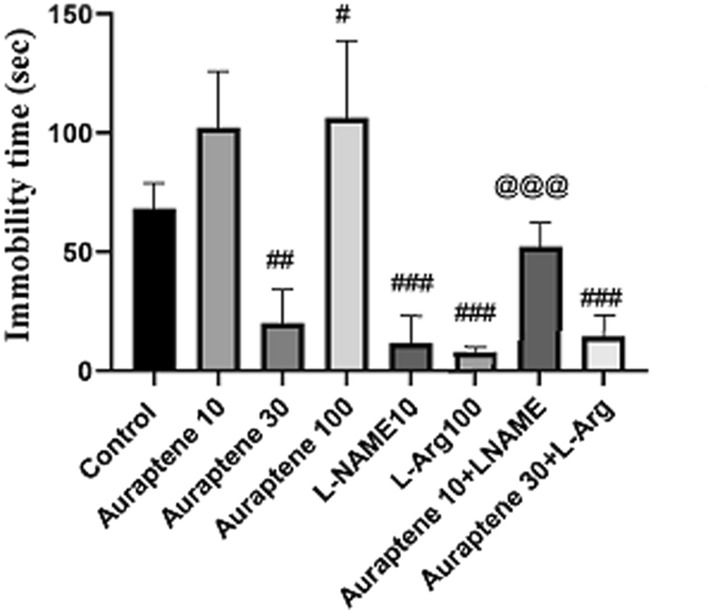
The duration of immobility in TST in the experimental groups. Data were reported as Mean ± SEM and analyzed using one-way ANOVA and Tukey post hoc test. ^#^ P < 0.05, ^##^P < 0.01, and ### P < 0.001 in compared to the normal group. ^@@@^ P < 0.001 in compared to the group that received Auraptene at a dose of 10 mg/kg

As illustrated in Fig. 4, the Administration of Auraptene (30 mg/kg) significantly reduced the duration of immobility in the TST compared to the normal saline group (P < 0.01). So the administration of Auraptene at 100 mg/kg significantly increased the duration of immobility compared to the normal saline group (P < 0.05). Also, administration of L-NAME (10 mg/kg) and administration of L-Arg (100 mg/kg) significantly reduced the duration of immobility compared to the normal saline group (P < 0.001). Based on the results, we observed that auraptene at a dose of 10 mg/kg did not exert an antidepressant-like effect compared to the normal saline-received group; thus, this dose was selected as a sub-effective dose and co-administrated with L-NAME. Moreover, findings showed that auraptene at a 30 mg/kg dose possessed an antidepressant-like effect; thus, we selected this dose as an effective dose and co-administrated plus L-Arg. Co-administration of Auraptene (30 mg/kg) and L-Arg significantly reduced immobility time compared to the normal saline group (P < 0.001). Administration of Auraptene (10 mg/kg) plus L-NAME significantly reduced the duration of immobility in the TST in comparison to the group that received 10 mg/kg Auraptene alone (P < 0.001).

### The duration of immobility in the FST

**Fig. 5:**
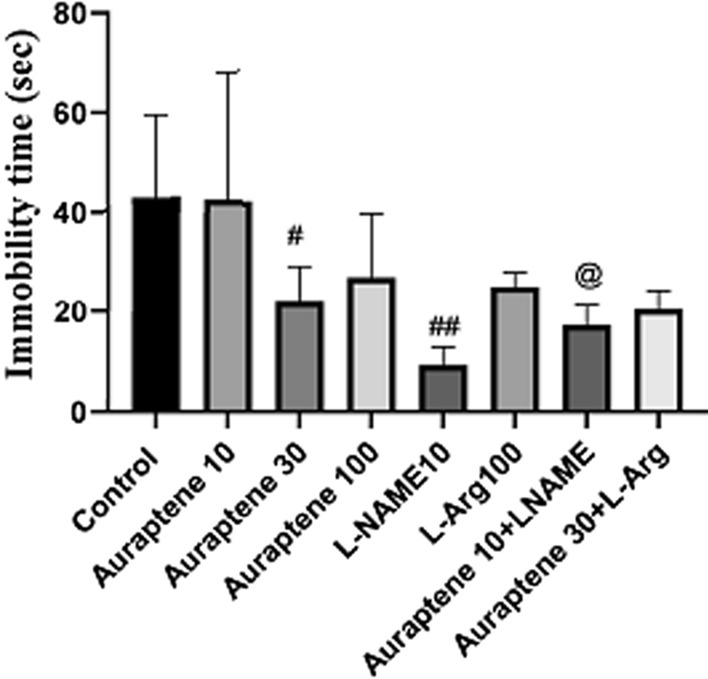
The duration of immobility in FST in the experimental groups. Data were reported as Mean ± SEM and analyzed using one-way ANOVA and Tukey post hoc test. ^#^ P < 0.05 and ^##^P < 0.01 in comparison to the normal group. ^@^ P < 0.05 in compared to the group that received Auraptene at a dose of 10 mg/kg

The results of this study (Fig. 5) showed that the administration of Auraptene at 30 mg/kg significantly reduced the duration of immobility in the FST compared to the normal saline group (P < 0.05). Moreover, administration of L-NAME (10 mg/kg) significantly reduced this time compared to the normal saline group (P < 0.01). Co-administration of Auraptene (10 mg/kg) and L-NAME significantly reduced the duration of immobility compared to the group that received 10 mg/kg Auraptene alone (P < 0.05).

### The serum and brain NO levels

**Fig. 6:**
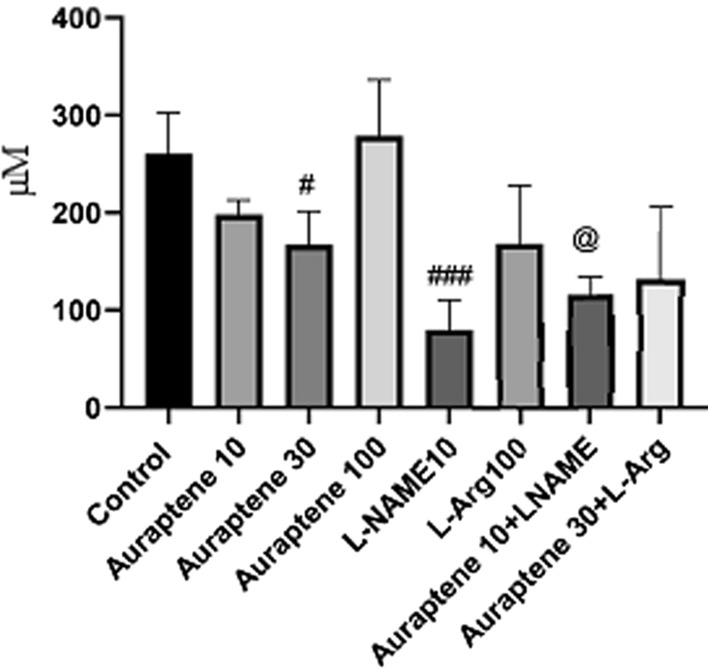
The serum nitric oxide levels (µM) in the different groups. Data were reported as Mean ± SEM and analyzed using one-way ANOVA and Tukey post hoc test. ^#^ P < 0.05 and ^###^P < 0.001 in compared to the normal group. ^@^ P < 0.05 in comparison to the group received Auraptene at a dose of 10 mg/kg

As illustrated in Fig. 6, administration of Auraptene at 30 mg/kg significantly reduced serum NO level compared to the normal saline group (P < 0.05), while doses of 10 and 100 mg/kg did not produce any significant difference in comparison with the normal saline group. Injection of L-NAME at 10 mg/kg significantly reduced serum NO level compared to the normal saline group (P < 0.001). Co-administration of Auraptene (10 mg/kg) and L-NAME significantly reduced the serum; NO level compared to the group that received Auraptene at 10 mg/kg (P < 0.05).

**Fig. 7:**
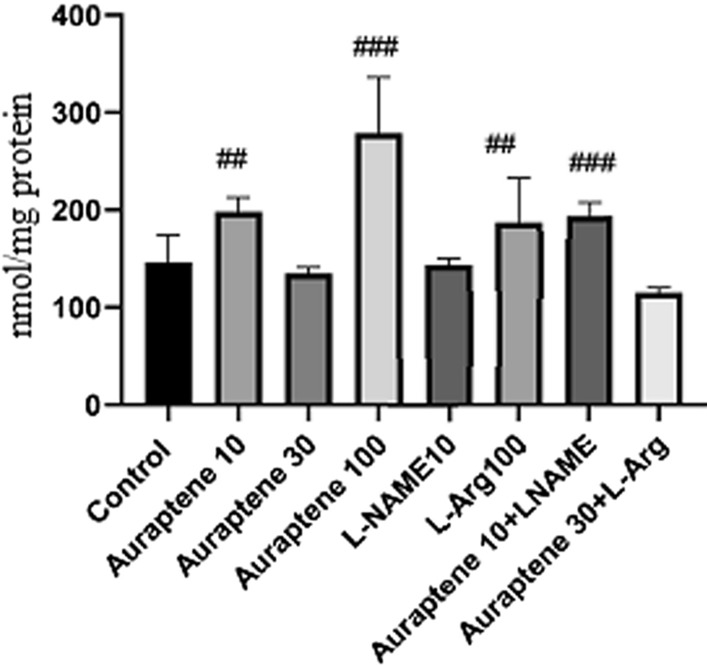
The study groups showed brain nitric oxide content (nmol/mg protein). Data were reported as Mean ± SEM and analyzed using one-way ANOVA and Tukey post hoc test. ^##^ P < 0.01 and ^###^P < 0.001 in compared to the normal group

According to the results (Fig. 7), administration of Auraptene at the doses of 10 and 100 mg/kg significantly increased the brain NO level compared to the normal saline group (P < 0.01 and P < 0.001). Besides, administration of L-Arg at 100 mg/kg significantly increased brain NO level compared to the normal saline group (P < 0.001). Co-administration of Auraptene (10 mg/kg) and L-NAME significantly increased this level compared to the normal saline group (P < 0.001).

### The serum and brain MDA levels

**Fig. 8:**
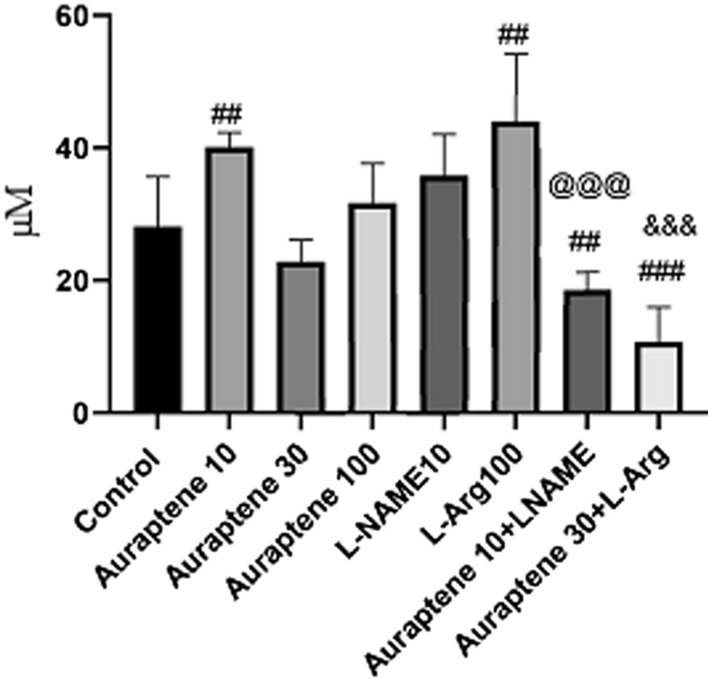
Serum MDA levels (µM) in the study groups. Data were reported as Mean ± SEM and analyzed using one-way ANOVA and Tukey post hoc test. ^##^ P < 0.01 and ^###^P < 0.001 in compared to the normal group. ^@@@^ P < 0.001 in compared to the group that received Auraptene at a dose of 10 mg/kg. ^&&&^P < 0.001 in compared to the group that received Auraptene at a 30 mg/kg dose

Tukey’s post hoc test (Fig. 8) showed that administration of Auraptene at 10 mg/kg significantly increased serum MDA levels compared to the normal saline group (P < 0.05). Co-administration of Auraptene (10 mg/kg) and L-NAME significantly reduced serum MDA levels compared to the normal saline group and the group that received Auraptene alone (P < 0.01 and P < 0.001, respectively). Moreover, administration of Auraptene (30 mg/kg) plus L-Arg significantly reduced serum MDA levels compared to the normal saline group and the group that received Auraptene alone (P < 0.001).

**Fig. 9:**
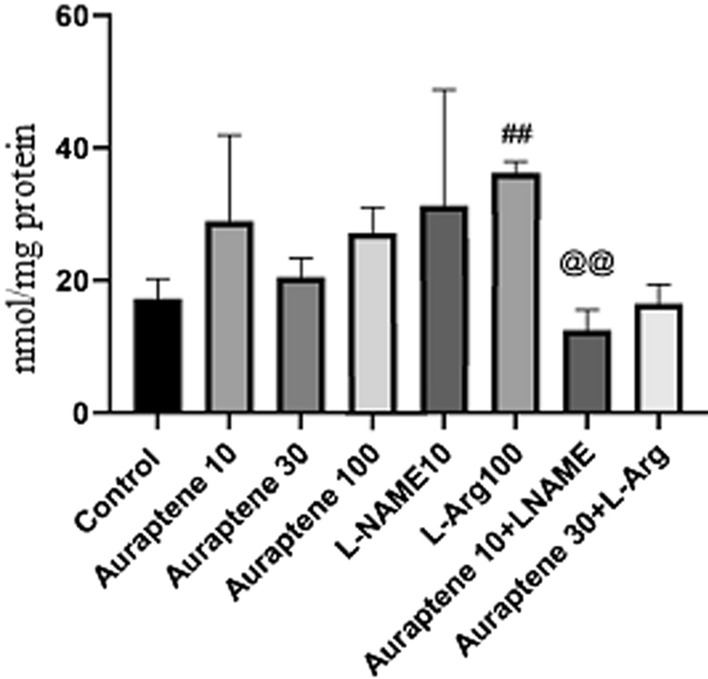
Brain MDA levels (nmol/mg protein) in the study groups. Data were reported as Mean ± SEM and analyzed using one-way ANOVA and Tukey post hoc test. ^##^ P < 0.01 in compared to the normal group. ^@@^P < 0.01 in compared to the group that received Auraptene at a dose of 10 mg/kg

The results (Fig. 9) showed that administration of Auraptene at 10, 30, and 100 mg/kg did not make any significant difference compared to the normal saline group. Nevertheless, the administration of L-Arg at 100 mg/kg significantly increased brain MDA levels compared to the normal saline group (P < 0.01). Co-administration of Auraptene (10 mg/kg) and L-NAME significantly reduced brain MDA levels compared to the group that received Auraptene at 10 mg/kg alone (P < 0.01).

### The serum and brain antioxidant capacity

**Fig. 10:**
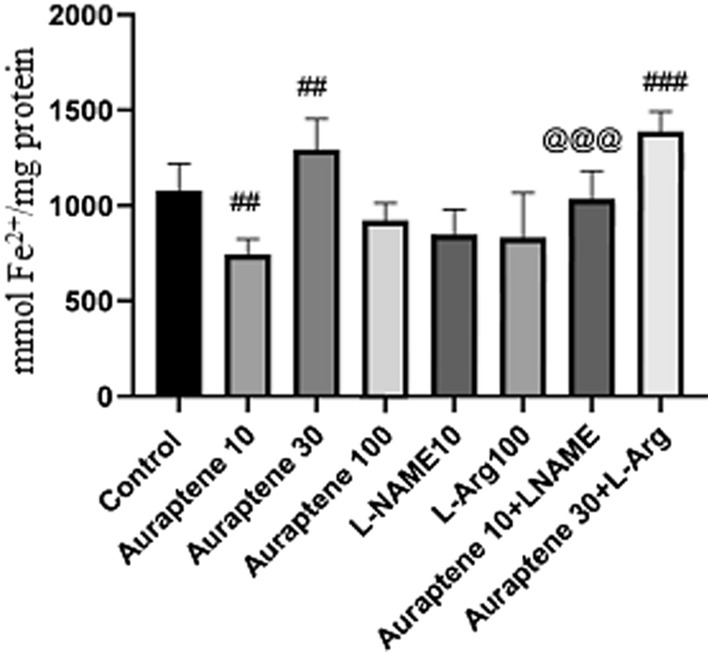
Serum antioxidant capacity (mmol Fe^2+^/mg protein) in the experimental groups. Data were reported as Mean ± SEM and analyzed using one-way ANOVA and Tukey post hoc test. ^#^ P < 0.05, ^##^P < 0.01, and ^###^P < 0.001 in compared to the normal group. ^@@@^ P < 0.001 in compared to the group that received Auraptene at a dose of 10 mg/kg

Administration of Auraptene at 10 mg/kg significantly reduced serum antioxidant capacity compared to the normal saline group (P < 0.001, Fig. 10). Auraptene at 30 mg/kg significantly reduced the serum capacity of antioxidants compared to the normal saline group (P < 0.01). However, at the 100 mg/kg dose, it did not have any significant difference. Injection of L-NAME at 10 mg/kg significantly reduced serum antioxidant capacity compared to the normal saline group (P < 0.05). Co-administration of Auraptene (30 mg/kg) and L-Arg significantly increased serum antioxidant capacity compared to the normal saline group (P < 0.001). Co-injection of Auraptene (10 mg/kg) and L-NAME significantly increased serum antioxidant level compared to the group that received Auraptene at 10 mg/kg alone (P < 0.001).

**Fig. 11:**
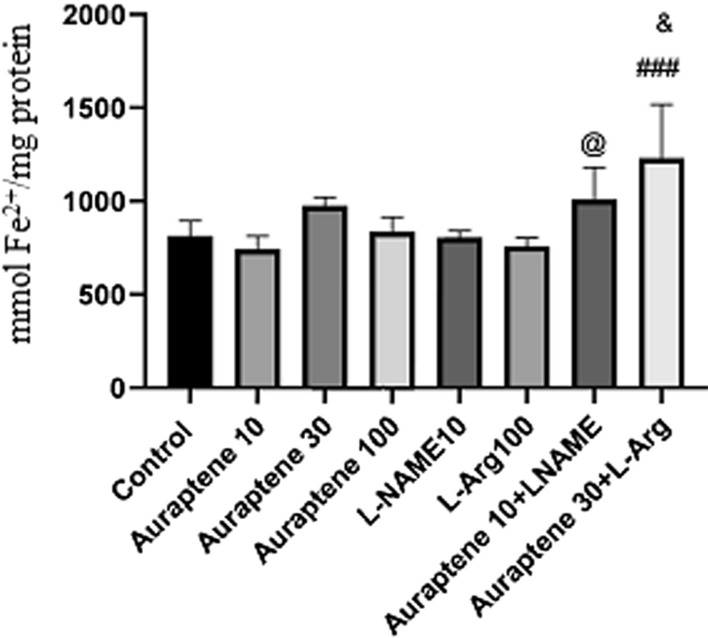
Antioxidant capacity of the brain (mmol Fe^2+^/mg protein) in the experimental groups. Data were reported as Mean ± SEM and analyzed using one-way ANOVA and Tukey post hoc test. ^###^ P < 0.001 in comparison to the normal group. ^@^P < 0.05 in comparison to the group received Auraptene at a dose of 10 mg/kg. ^&^ P < 0.05 in compared to the group that received Auraptene at a 30 mg/kg dose

Figure 11 illustrates no significant difference in the brain antioxidant capacity between the groups that received Auraptene at doses of 10, 30, and 100 mg/kg and the normal saline group. But the administration of Auraptene (30 mg/kg) plus L-Arg significantly increased brain antioxidant capacity compared to the normal saline group, and the group received 30 mg/kg of Auraptene (P < 0.001 and P < 0.05 respectively). Besides, co-administration of Auraptene (10 mg/kg) and L-NAME significantly increased the level of antioxidants in the brain is compared to the group that received 10 mg/kg Auraptene alone (P < 0.05).

## Discussion

This study aimed to investigate the antidepressant-like effect of Auraptene in male mice with regards to the NO pathway. Our results showed that Auraptene reduced the immobility duration in the FST and TST, indicating its antidepressant-like effect. We showed that Auraptene partially, at least, via an increase in antioxidant capacity and the reduction in the MDA and nitrite levels in the brain possessed its antidepressant-like effects.

It has been demonstrated that depression in rodents is associated with increased immobility time in the FST and TST [30]. Preclinical studies have shown that agents with antidepressant-like effects decreased the immobility time in the FST and TST [31]. This study observed that Auraptene reduced immobility time in the FST and TST, indicating its antidepressant-like effects.

Auraptene (7-geranyloxycoumarin) is a coumarin derivative found in many plants of the citrus genus, such as grapefruit and oranges [13]. Various pharmacological properties, including anti-cancer, antibacterial, antifungal, anti-inflammatory, and antioxidant effects, have been reported for Auraptene [15–17, 32]. A randomized, placebo-controlled, double-blind study in healthy volunteers showed that consumption of Auraptene as peels of citrus Kawachiensis (Kawachibankan) improved cognitive function [33]. Experimental studies have been determined that Auraptene has neuroprotective properties. In this regard, it has been shown that Auraptene, probably through attenuation of oxidative stress, exerted an anticonvulsant effect in pentylenetetrazol-induced chemical kindling in mice [34]. Furthermore, Auraptene mitigated nerve damage and enhanced memory following cerebral ischemia [35]. Our results showed that Auraptene possessed antioxidant properties that increased antioxidant capacity and reduced MDA and nitrite levels in the brain. These properties are contributed to possibly the antidepressant-like effect of Auraptene.

It has been determined that nitric oxide (NO) is involved in the pathophysiology of behavioral disorders like depression [36]. In this concept, previous studies showed that NO overproduction mitigated the antidepressant effect of antidepressants [37]. Moreover, inhibition of NO production potentiated the antidepressant effects of some agents [38, 39]. In agreement with the abovementioned studies, our findings showed that NOS inhibitor (L-NAME) potentiated the antidepressant-like effect of a sub-effective dose of Auraptene. At the same time, L-arg (a NO precursor) attenuated the antidepressant-like effect of the effective dose of Auraptene. Therefore, possibly, NO is likely to contribute to the antidepressant-like development of Auraptene.

Clinical studies have demonstrated a direct association between concentrations of MDA in the plasma with the severity of depressive symptoms [40]. In this respect, researchers reported that major depressive disorder is associated with a decrease in antioxidant status and induction of oxidative and nitrosative pathways [6, 41, 42]. It has been well-determined that reduced antioxidant capacity is associated with increased MDA levels [43]. Ample evidence showed that some antidepressants exerted their effects by increasing the antioxidant capacity and decreasing MDA level [44, 45]. Our results showed that Auraptene significantly decreased MDA and increased antioxidant capacity. Furthermore, we observed that co-administration of L-NAME potentiated and L-arg mitigated the effect of Auraptene on the MDA level and antioxidant capacity.

This study indicates that Auraptene possessed an antidepressant-like effect by reducing NO and MDA levels and increasing antioxidant capacity. However, the mechanisms of action of Auraptene based on our study are not entirely determined, so; further studies are necessary to find exact mechanisms involved in the antidepressant-like effect of Auraptene.

## Conclusions

Our results indicate that Auraptene exerted antidepressant-liker activity in mice by decreasing the NO and MDA levels and increasing antioxidant capacity. Findings showed that beneficial effects of Auraptene on depressive-like behaviors and oxidative stress markers are mediated via the NO pathway. Furthermore, we found that NOS inhibition potentiated while NO precursor mitigated the beneficial effects of Auraptene.

## Data Availability

Data regarding the present study are available at Medical Plants Research Center, Shahrekord University of Medical Sciences.

## Abbreviations

NO: Nitric oxide
MDA: Malondialdehyde
L-NAME: L-Nitro arginine methyl ester
NA: Noradrenaline
DA: Dopamine
nNOS: Neuronal nitric oxide synthase
iNOS: Induced nitric oxide synthase
MAO-A: Monoamine oxidase A
OFT: Open field test
TST: Tail suspension test
FST: Forced swim test
NaOH: Sodium hydroxide.
